# Asymptomatic *Plasmodium falciparum* infections and determinants of carriage in a seasonal malaria chemoprevention setting in Northern Cameroon and south Senegal (Kedougou)

**DOI:** 10.1186/s12936-024-05150-3

**Published:** 2024-12-18

**Authors:** Innocent M. Ali, Isaac A. Manga, Akindeh M. Nji, Valery P. Tchuenkam, Peter Thelma Ngwa Neba, Dorothy F. Achu, Jude D. Bigoga, Babacar Faye, Cally Roper, Colin J. Sutherland, Wilfred F. Mbacham

**Affiliations:** 1 MARCAD Programme, The Biotechnology Centre, University of Yaoundé 1, Yaoundé, Cameroon; 2 Department of Biochemistry, University of Dschang, Dschang, Cameroon; 3 Department of Parasitology and Mycology, Faculty of Medicine and Odontostomatology, Université Cheick Anta Diop, Dakar, Senegal; 4 Department of Infection Biology, Faculty of Infectious and Tropical Diseases, London School of Hygiene & Tropical Medicine, London, UK; 5 National Malaria Control Programme, Ministry of Public Health, Yaoundé, Cameroon

**Keywords:** Seasonal malaria chemoprevention, Asymptomatic malaria, Adamaoua, Kedougou, Plasmodium

## Abstract

**Background:**

Among the several strategies recommended for the fight against malaria, seasonal malaria chemoprevention (SMC) with sulfadoxine-pyrimethamine and amodiaquine combination (SPAQ) targets children 3 months to 5 years in Sahel regions of Africa to reduce mortality and mortality. Since SMC with SPAQ is administered to symptoms-free children for prevention of malaria, it is anticipated that a proportion of asymptomatic parasitaemic children will also be treated and may result in a drop in both the overall population prevalence of asymptomatic malaria infections, subsequent risk of symptomatic malaria infections and transmission. Age-specific carriage of asymptomatic *Plasmodium* spp. infections (API) was evaluated in target children and adults in Cameroon and Senegal, prior to the 2018 SMC campaign in both countries.

**Methods:**

A baseline household survey was carried out in August 2018 in two areas in Cameroon and one in Senegal just before the beginning of distribution of SPAQ for SMC. The survey included collection of fingerpick blood for malaria rapid diagnostic testing (RDT) and administration of a pre-tested questionnaire on demographics and malaria risk factors to participants. The age-specific prevalence of API in all study sites was analysed, first as a distribution of RDT-positives in 5-year age categories and secondly, with age as a continuous variable in the whole sample, using the Wilcoxon rank sum test. Risk factors for carriage of asymptomatic infections were examined using logistic regression analysis in STATA v.16 and Rv4.1.2.

**Results:**

In total, 6098 participants were surveyed. In Cameroon, overall prevalence of API was 34.0% (32.1–36.0%) in Adamaoua, and 43.5% (41.0–45.7%) in the North. The median age of RDT positivity was higher in Senegal: 11 years (IQR 7–16) than in Cameroon—Adamaoua: 8 years (4–17) and North: 8 years (4–12) and significantly different between the three study regions. In all three study sites, asymptomatic carriage was significantly higher in the older age group (5–10 in Cameroon, and 7–14 in Senegal), compared to the younger age group, although the median age of participants was lower among RDT-negatives in the North compared to RDT-positives. Health area, gender and last infection within past year significantly confounded the relationship between age and parasite carriage in Adamaoua and Senegal but not in North Cameroon. Absence of bed net and previous infection within one month of the survey all independently predicted carriage of asymptomatic parasites in multivariate regression analysis.

**Conclusion:**

Under five years asymptomatic *Plasmodium* infection in northern Cameroon prior to SMC season remained high in 2018, irrespective of history of SMC implementation in the study areas in Cameroon. Compared to Adamaoua, peak asymptomatic malaria parasite rate was observed in children 5–10 years, which is out of the SMC target age-range. Health area, last infection within the past month and to a lesser extent gender affected the association between age and asymptomatic carriage in all sites except the North region of Cameroon, indicating wide heterogeneity in risk of malaria among the general population in that geography. Follow-up studies designed to measure SMC effects in Cameroon are warranted as it may become necessary to extend age of SMC eligibility to 10 years, as is practiced in Senegal.

## Background

Malaria is a recurrent public health challenge in sub-Saharan Africa where the burden of disease remains high, despite interventions that markedly reduced the global malaria burden since 2000 [1]. An upsurge in the disease was recently reported by the World Health Organization (WHO) [2]. Among the people living in endemic areas, a significant proportion of people asymptomatic infections due to non- immunity that develops following exposure to infected mosquito bites [3]. Asymptomatic carriers represent an important reservoir for malaria transmission as the generation of parasite transmission stages continues during prolonged sub-clinical infection [4, 5]. Therefore, interventions that envisage malaria elimination should include targeting of parasites by asymptomatic individuals, as changes in prevalence of these will be an important indicator of the effectiveness of such interventions.

Among the several strategies recommended by the WHO for reducing the malaria burden, seasonal malaria chemoprevention (SMC) has been shown to provide effective protection for children 3 months to 5 years in Sahelian regions of Africa [6]. This strategy consists of repeated administration of full doses of sulfadoxine-pyrimethamine plus amodiaquine (SPAQ) to these children monthly for three to four months representing the high transmission period [7]. The goal is to reduce morbidity and mortality in the vulnerable group during this period. Because SPAQ is administered to children without symptoms for prevention of subsequent clinical malaria, it is anticipated that a reduction in parasite carriage in this group may also result in a drop in both the overall prevalence of asymptomatic and symptomatic infections and transmission of malaria in the general population.

In the Sahel areas of Senegal (south) and Cameroon, SMC has been implemented as a strategy to reduce malaria mortality and morbidity since 2015 and 2016, respectively. A recent report by the National Malaria Control Programme (NMCP) in Cameroon indicates that after the first year of SMC implementation, more than 1.5 million children 3 months–5 years received SPAQ with a coverage of 88% after 3 rounds of administration of SPAQ (NMCP Summary SMC Implementation Report, 2017). In Senegal, SMC implementation was stepwise, following a step-wedged randomized control trial of the intervention in eligible districts in the country and included children under 5 years of age initially, extending to 10 years following preliminary information indicating substantial burden of malaria in 5–10 years age group [8].

No systematically collected information is available on the community burden of asymptomatic malaria in regions of SMC implementation in Cameroon, and knowledge of other key indicators, such as rates of transmission stages and parasite drug resistance among asymptomatic infections is also lacking. In this study, the baseline prevalence of RDT-confirmed asymptomatic *Plasmodium falciparum* infections among both adults and children less than 5 years in Cameroon and Senegal was assessed. The distribution of RDT positivity across the population and between under-fives and older age groups was then studied. Parasite carriage was compared among children less than five and adults in the North, with a history of SMC and the ecologically similar Adamaoua region, where SMC had not yet been implemented. The current report represents results of a baseline prevalence of asymptomatic *P. falciparum* infections before the SMC campaign of 2018. It is part of a larger study investigating markers of drug resistance within a cohort of children less than 5 years in SMC areas in Cameroon and Senegal. This represents one of the few studies that describe asymptomatic *P. falciparum* infections in a seasonal malaria chemoprevention setting and a neighbouring setting with no history of SMC implementation. Further, it provides insights into how baseline asymptomatic parasite carriage varies with age until at least 40 years, highlighting the need for countries such as Cameroon to consider extending age eligibility for delivery of seasonal malaria chemoprevention to around ten years.

## Methods

### Study setting and population

**Fig. 1:**
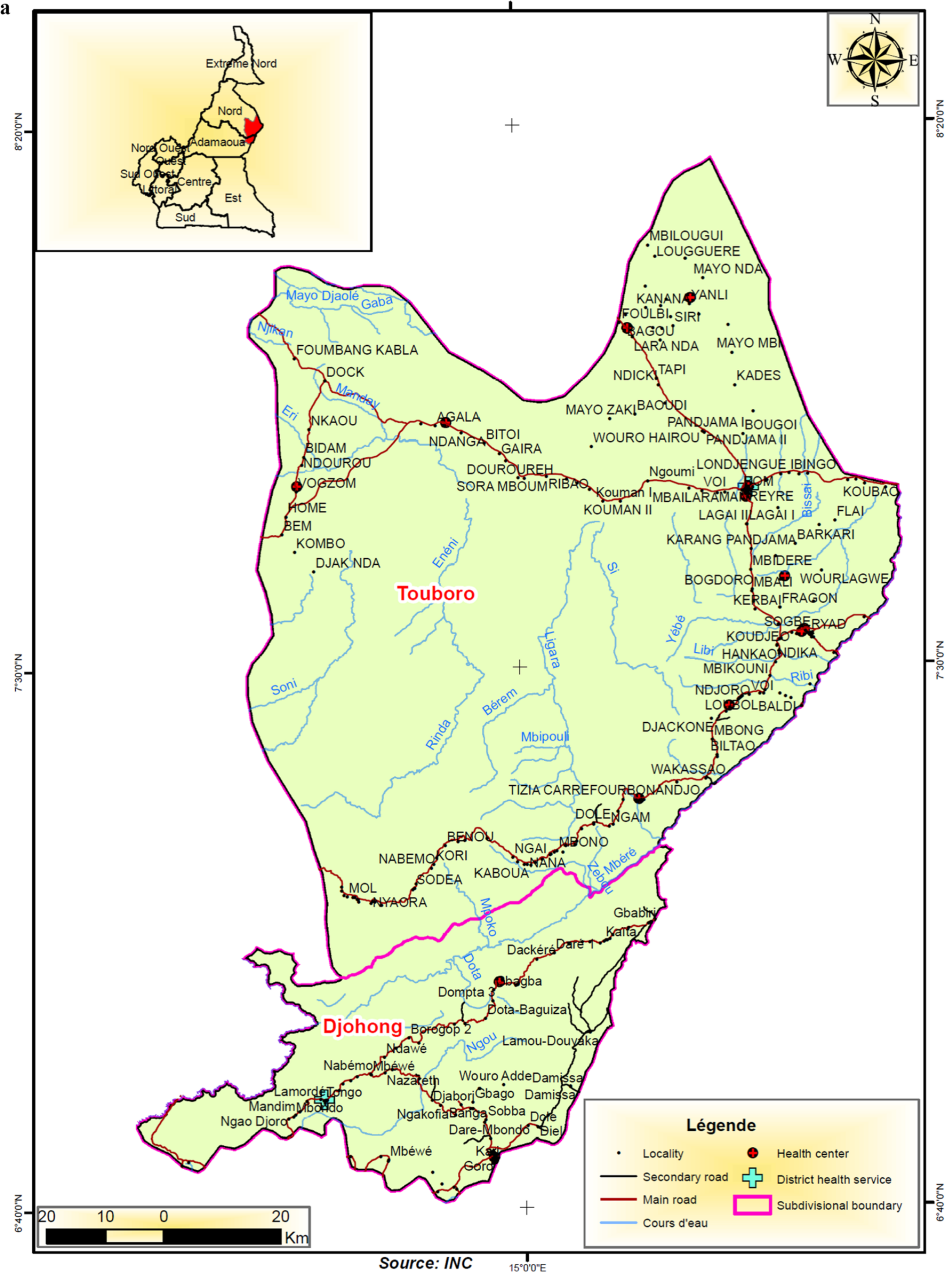

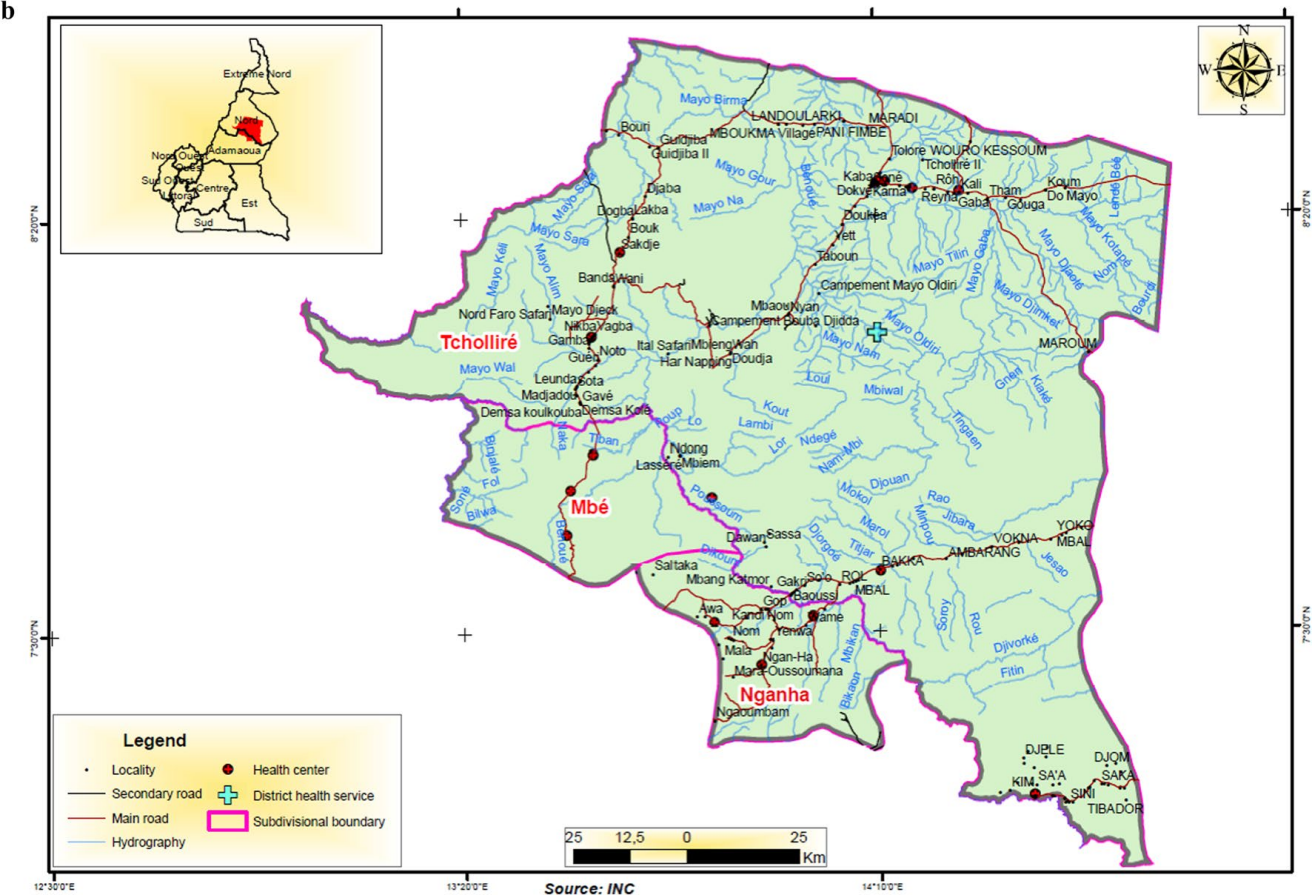

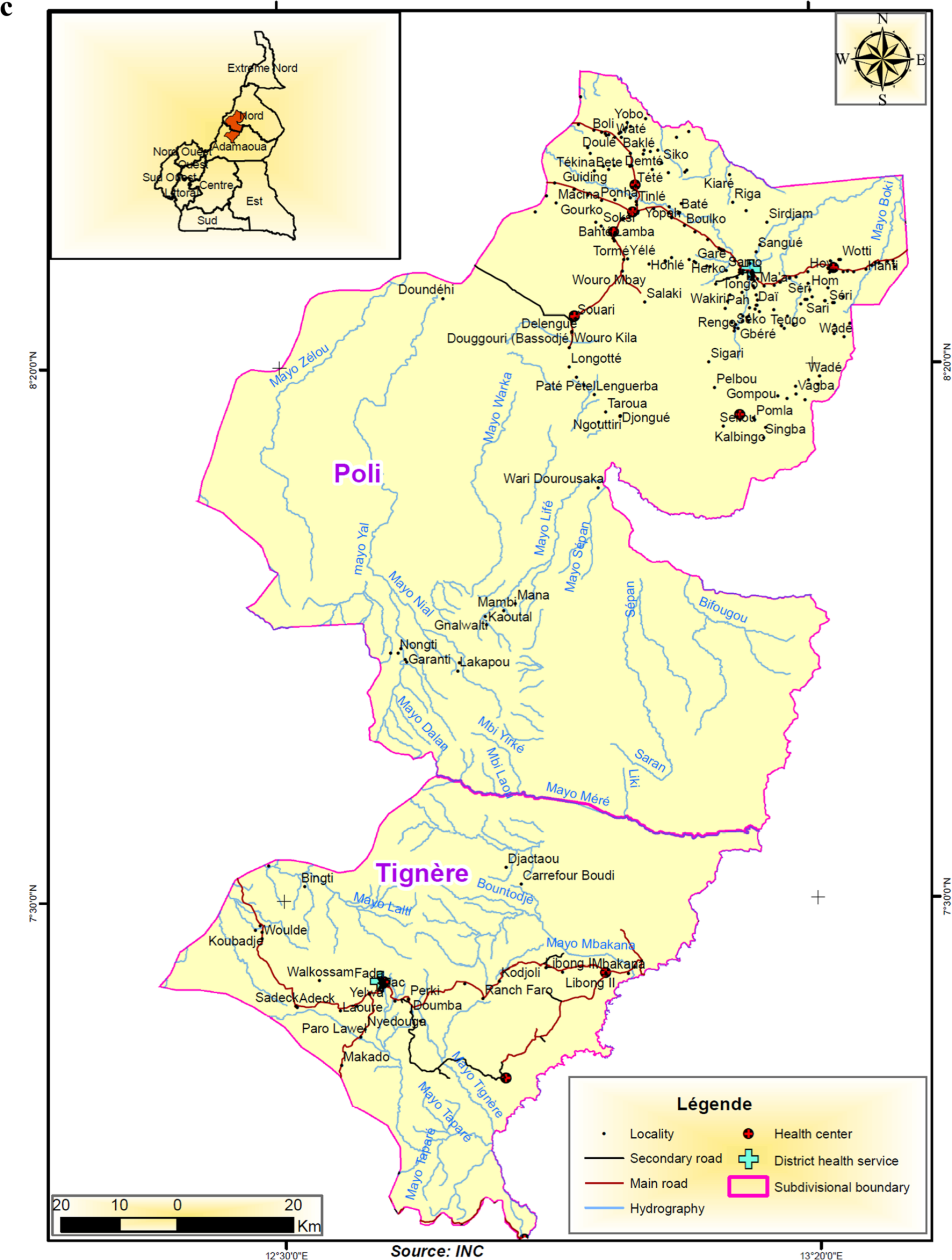

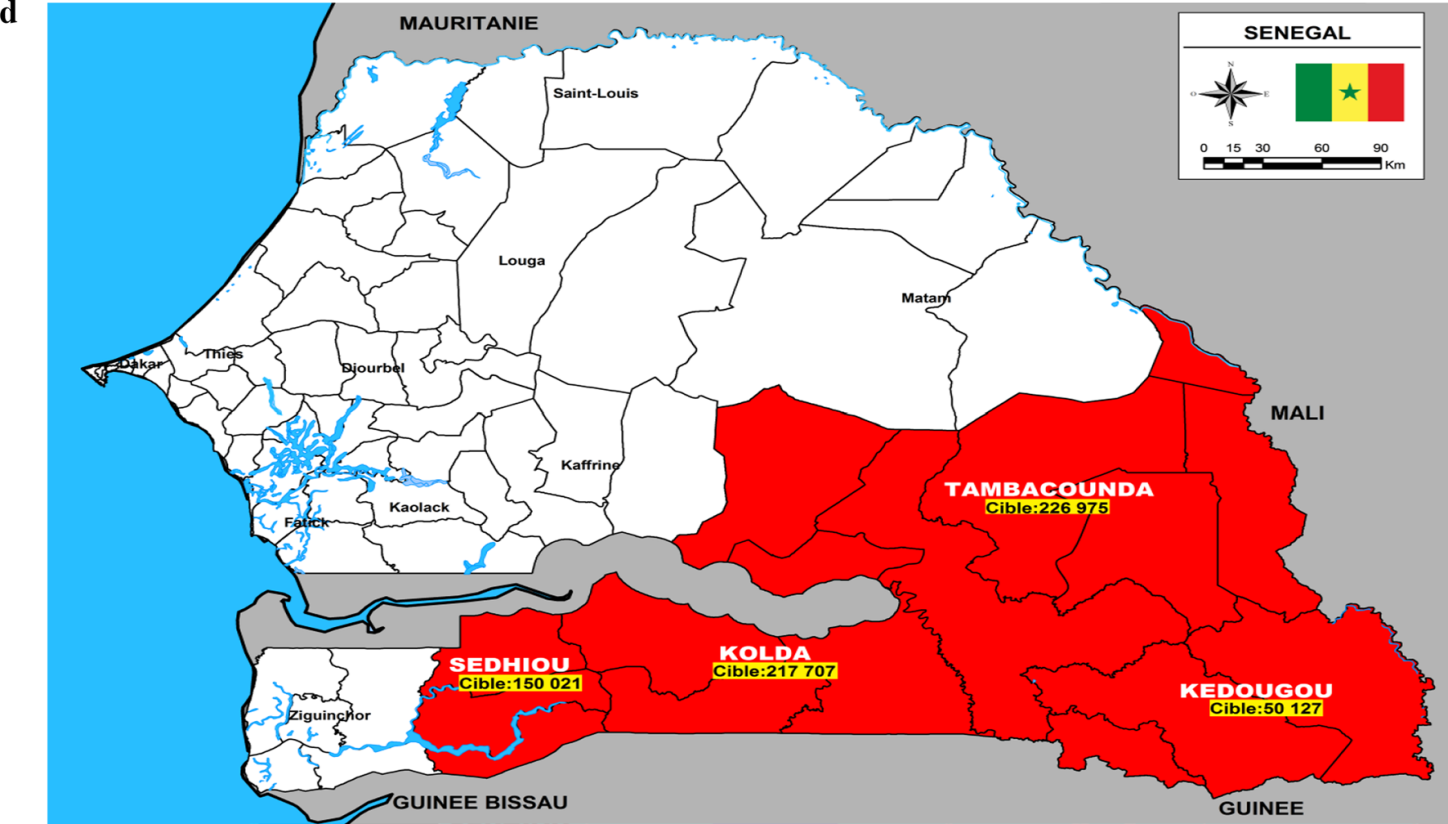
**A** Location of study areas in western Adamaoua and Nord Regions of Cameroon. **B** Location of study areas in central Adamaoua and Nord Regions of Cameroon. **C** Location of study areas in eastern Anandamou and Nord Regions of Cameroon. **D** A Map of Southern Senegal indicating the region where SMC is practiced. Regions in red represent localities that were included in the study

This study took place in 10 health districts in northern Cameroon and southern Senegal. In Cameroon, two regions representing six health districts were chosen. Four of the districts are located in the North region and share a boundary with the rest of the districts located in the Adamaoua region (Fig. 1A–C). The Adamaoua region as a whole is not eligible for yearly implementation of seasonal malaria chemoprevention with SPAQ because the requirement for highly seasonal malaria transmission 3–4 months per year is not met in most districts. However, the selected health districts in Adamaoua sharing boundaries with the North region have similar ecological characteristics to the North region and although potentially suitable for SMC implementation, this has not occurred. In the North region, health areas in the Tchollire, Poli, Rey Bouba and Touboro health districts were included in the survey while in the Adamaoua region, health areas in the Ngaoundere rural, Tignere and Djohong health districts were included.

Access to two health areas located in Djohong and Touboro health districts was limited by security concerns and our randomization was, therefore, limited to those eligible districts deemed safe to visit. In Fig. 1A–D, maps of the study areas are shown, including the catchment areas of health centres, villages, hydrography (principally seasonal river courses that exist mainly in the wet season), secondary and primary roads. In Senegal, health areas in the Kedougou and Kolda regions, particularly in the health district of Saraya in Kedougou and of Velingara in Kolda were included in the survey.

### Sampling design and population

This study was a cross-sectional household survey enrolling 6093 male and female adults and children of which 1773 were from southern Senegal and 4320 from Cameroon (2080 from North and 2240 from Ngaoundere). This involved a total of 32 villages in Cameroon and 10 from southern Senegal. The villages were selected using probability proportional to estimated size (PPS) based on survey carried out prior to the distribution of insecticide-treated bed nets in 2016 in two health districts by the NMCP. Large villages were segmented to contain approximately the same number of children. In each village/segment selected randomly, all households were eligible to participate. Houses where the head of household did not consent to participation were not replaced. Temporary visitors were excluded in the survey. The needs of the NMCP, responsible for conduct of SMC campaigns in the communities as part of their mandated routine, were always maintained as the priority during the study.

### Recruitment of participants

Prior to deploying study personnel to the field, sensitization was undertaken in each community. Social mobilization included using prominent leaders of community health workers and existing focal persons for childhood vaccination advocacy in the area. Since this survey took place at the start of the administration of SMC for 2018 in all regions, it was important to adequately inform communities on the purpose of the survey, when the campaign would start, how the survey relates to on-going SMC and the importance of adherence to on-going SMC schedules. In Cameroon, community mobilization is done 3–4 days prior to distribution and administration of SPAQ in the SMC programme. The study was conducted during these days including the first day of drug administration to ensure that children under five years of age whose parents provided consent for the study were eligible to participate. Participants in selected households were approached by Research Assistants.

SMC eligibility in Cameroon is defined by child (3–59 months old) being asymptomatic on the first day of administration of SPAQ and absence of any other symptom or underlying pathology requiring urgent medical attention. ‘Asymptomatic’ was defined as absence of fever (and history of fever in the past 24–48 h) or any other symptom suggestive of malaria (lethargy, shivering, loss of appetite, drowsiness) on the day of distribution of SPAQ to eligible households (having at least one eligible child). Absence of fever on the day of sampling was ascertained by measuring axillary temperature; which is the displayed temperature on the digital thermometer plus the correction factor (0.5); and was < 37.5 °C. History of fever was reported by the caregiver when prompted. As standard practice, children are not screened before being offered a course of SPAQ for SMC. Symptomatic adults and children were referred to the community health worker or to the nearest health centre as appropriate.

Based on the design of the study, all consenting children and adults in selected enumeration area were sampled. Bed net presence/state was reported by the head of the household and viewed/examined by the Research Assistant. Bed net use in the past 24 h was reported by the head of household (mother). Children and adults who were afebrile and RDT positive were not treated, but the head of the household was encouraged to refer to community health workers or visit the nearest health centre in case symptoms develop. This technique was efficient because in each enumeration/health area, the supervisor of the survey was also the head of the health facility. The approach taken was part of the approved protocol by the Cameroon National Ethics Committee prior to implementation. Written consent/assent was sought from members of each household after the head of the household consented. Those who did not consent/or assent were excluded from further research activities. Participants were attributed unique study codes and administered a short questionnaire by Research Assistants using a personal digital assistant (PDA) that contained a simple questionnaire designed to capture participant demographics, history of fever, previous diagnosis and treatment for malaria and questions around malaria prevention measures.

### Blood sampling and laboratory testing

Finger stick blood was collected from each participant and used for RDT testing immediately based on manufacturer’s instructions. The same participant code was used for all diagnostics and maintained through all laboratory procedures. Field workers collecting blood were trained to perform finger stick, implement RDT testing protocol, interpret RDT test results, record in PDAs and waste disposal/biosafety. The RDT test used could simultaneously detected two key antigens in *Plasmodium*: Histidine Rich Protein 2 specific for *P. falciparum* and parasite lactate dehydrogenase secreted by all other malaria parasite species detected by the RDTs.

### Data management and statistical analysis

#### Sample size calculation

The primary outcome for this study was the age-specific prevalence of asymptomatic *P. falciparum* infection in children 3 months to 5 years versus the rest of the population. It was assumed that 18% of the population was made of children of the desired age and who are eligible for SMC. The population size of each village unit selected was expected to have on average 56 eligible children and it was planned, therefore, to sample 40 village units to give 2464 participants adjusted for a 10% non-response rate based on past experience. Assuming an average prevalence of API among over 5 s of 30%, and a ratio of 1:2 of under 5 s to older age groups in our surveys, this sample size provides 80% power at the 5% significance level to detect a lower prevalence among under 5 s of 24.6%, representing a delta of 5.4%.

#### Outcomes and variables

Data from questionnaires were downloaded from PDAs as an Excel file and cleaned. Queries were resolved with supervisors of field teams in both countries. Questionnaire data collection was done before RDT testing by one of the field workers who was also blind to RDT test outcome that was recorded by the second field worker. The response rate was calculated as the proportion of consented participants out of all eligible participants in all households visited. The primary outcome of this survey was RDT confirmed asymptomatic *P. falciparum* infection among children 3 months to < 5 years in the study settings in Cameroon and Senegal. Secondary outcomes were (i) an age distribution of asymptomatic carriage of *P. falciparum* in the study populations in Cameroon and Senegal, with age treated as a continuous variable; (ii) risk factors associated with asymptomatic carriage in children less than five years in each site. Bed net characteristics, ownership and use in the study populations prior to the beginning of SMC round of that year were also described in each geography.

#### Statistical methods

The prevalence of asymptomatic *P. falciparum* carriage was estimated as the proportion of asymptomatic malaria parasite infections by RDT positivity in the entire population of tested participants in each study site. The Chi-square test was used to compare the prevalence of asymptomatic malaria parasite infections between communities, generating odds ratios and 95% confidence intervals (95% CI). The distribution of RDT positivity among the study population was then analysed with age as a continuous variable using the non-parametric Wilcoxon rank sum test. A multivariate analysis was conducted using logistic regression of the main variable “RDT outcome” with two modalities, coded 1 = RDT + for asymptomatic *P. falciparum* infection and 0 = RDT- no asymptomatic *P. falciparum* infection and the effect of confounders on the relationship between RDT positivity and age examined. The model fit was tested by first by performing diagnostic checks for multicollinearity and variance inflation by predictors with tolerance value for all predictors set at p > 0.2. The adjusted odds ratios are presented. In all assessments, a P value less than 5% was considered significant and adjusted odds ratios interpreted alongside their confidence interval. R (v4.2.2 R Core Team, 2022) and STATA statistical software (STATA Release 16(2019), StataCorp LLC, College Station, TX) were used for all the analyses.

### Ethical considerations

This research protocol was reviewed and ethical clearance provided by the National Ethics Committees of Cameroon and Senegal (000179/MSAS/DPRS/CNERS of 22 December, 2017 for Senegal and 2018/01/961/CE/CNERSH/SP of 04 January, 2018). Administrative approval from the Minister of Public Health in Cameroon was also provided. Study participants provided written consent (≥ 21 years) or assent (< 21 years) and parental authorization (in the case of infants and young children) for all study procedures after understanding and agreeing on the purpose, objectives, procedures, risks and benefits of the study. All study-related information was stored securely at the investigator facilities. All laboratory specimens, data collection instruments, and administrative forms were identified by a coded number to maintain confidentiality. The de-identified data was used for analysis and preparation of other material for dissemination. The study was conducted in full compliance with the approved protocol. Compliance was maintained through training and supervision of qualified field team members (nurses and laboratory technicians) on all study procedures. Children and adults who were febrile during the household survey were referred to the nearest health facility or community health worker trained to diagnose and treat simple malaria in the area. Afebrile participants who had a positive RDT test result were not treated for malaria, but were advised to visit the community health worker or the nearest health centre if fever developed at any point in time after our visit. In each community, the study team included a respected local health facility worker to further safeguard against any form of exploitation of study participants in the community by study team members or by the participant community.

### Patient and public involvement

The protocol was developed with inputs from the NMCP based on needs in the communities. The National Malaria Control Programmes of Cameroon and Senegal implement SMC as part of their yearly routine for malaria control in the northern parts of Cameroon and southern parts of Senegal with highly seasonal malaria transmission. The NMCP are in close contact with the community and study participants. Patients were not involved in this study since it was community based and included symptom-free children and adults. Community mobilization was done using community health workers some of whom became study participants by reason of the statistical selection procedure. The results of this study were shared with the NMCP managers and northern regional malaria control implementers as well as in a poster presentation at the American Society of Tropical Medicine and Hygiene in the USA.

## Results

### Demographic characteristics

Table 1 presents the socio-demographic composition of the study population in sites in Cameroon and Senegal. In total, 4172 participants (2084 participants in North Cameroon, 2234 in Adamaoua) in Cameroon and 1774 participants in Kedougou were included in the study. Participants were sampled from 15 health areas (8 in Ngaoundere and 7 in the North) in Cameroon and 5 health areas in Kedougou, Senegal. In Cameroon, access to one health area in the North was limited by security concerns. There were slightly more females than males in all the sites with a female to male ratio of 1.2, 1.1 and 1.2 in Senegal, Adamaoua and North respectively. In the sample, children less than 5 years represented 22.9% in Kedougou, Senegal, 31% in Adamaoua and 40.2% in North Cameroon.

**Table 1:** Socio-demographic characteristics of the study population in two regions of Cameroon and in south Senegal (Kedougou)

Site	Variable	Modality	n	%
Kedougou, Sénégal	Sex	Missing	1	
		F	973	54.8
		M	800	45.1
	Age	Missing	89	
		≤ 5	385	22.85
		> 5	1300	77.15
	Health area	Bembou	595	33.23
		Kalifourou	305	17.27
		Kandia	592	33.82
		Sabodala	174	09.55
		Wadiyatou	107	06.11
Adamaoua, Cameroon	Sex	F	1185	52.9
		M	1054	47.1
	Age	≤ 5	689	30.8
		> 5	1551	69.2
	Health Area	Wack	98	4.4
		Tignere	648	28.9
		Nga1	148	6.6
		Mbe	400	17.9
		M’Mboum	448	20
		Gajiwan	99	4.4
		G. Yelwa	99	4.4
		Djohong	300	13.4
Nord, Cameroon	Sex	Missing	3	
		F	1151	55.31
		M	930	44.69
	Age	Missing	4	
		≤ 5	838	40.21
		> 5	1246	59.79
	Health Area	Gamba	160	7.68
		Hormbali	120	5.76
		Kali	160	7.68
		Mbaka	301	14.44
		Poli	560	26.87
		Sakje	480	23.03
		Tcholire	303	14.54

### Bed net ownership, state and use

Bed net ownership was high in all study areas (Table 2). However, there was evidence of reported use the previous night being below 80% in Kedougou, Senegal and Ngaoundere, and of nets in poor condition being retained longer than recommended in both Cameroon sites compared to the Senegal site. Less than 2.5% of participants reportedly spent the night out of home in the past two weeks following the survey in the North of Cameroon and in South of Senegal while up to 10.6% reportedly slept out of home in Ngaoundere, Cameroon.

**Table 2:** Bednet characteristics, ownership, use in the the study sites

Variables	Modalities	Senégal (N = 1774)		Adamaoua (N = 2240)		Nord (N = 2084)	
		n	%	n	%	n	%
Presence of bednet	Yes	1669	94.1	1908	85.2	1861	89.2
	No	105	5.9	332	14.8	211	10.14
	Don’t know	–	–	–	–	242	11.63
Type of bednet	LLIN	1550	99.55	1852	82.7	1844	88.5
	Other	–	–	16	0.7	14	0.7
	Don’t know	4	0.44	372	16.6	222	10.67
Slept under Bednet last night	Yes	1271	73.91	1655	73.9	1751	84
	No	502	26.08	266	11.9	109	5.2
	Don’t know	–	–	319	14.2	220	10.6
Bednet Bound to Mattress	Yes	1522	93.96	1494	66.7	1787	85.91
	No	108	06.03	419	18.7	51	2.45
	Don’t know	–	–	327	14.6	242	11.63
Bednet in good State	Yes	1245	76.90	821	41.2	1780	85.57
	No	398	23.09	892	44.8	55	2.64
	Don’t know	–	–	278	14.0	245	11.78
Duration since having Bednet	≤ 2 ans	1378	89.41	303	13.5	49	2.4
	> 2 ans	206	10.58	1596	71.3	1683	80.8
	Don’t know	–	–	340	15.2	348	16.7
Slept out of home	Yes	46	02.38	238	10.6	50	2.4
	No	1710	97.61	1949	87	1987	95.3
	Missing	–	–	52	2.3	44	2.1

Bednet characteristics were assessed in the study when bender was present. The state of the bender was assessed by counting the number of holes. When 5 or more, state was no. Slept out of home was assessed by asking if within past two weeks participant slept out of their home

### Age distribution and under 5 carriage of asymptomatic *Plasmodium* infections in Cameroon and Senegal

**Fig. 2:**
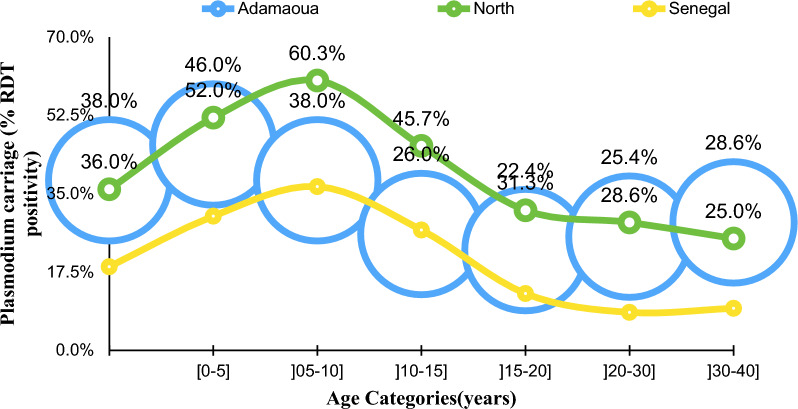
Variation in RDT positivity by age band among the study population up to 40 years. It should be noted the age of SMC eligibility is under 5 years. Clearly, the distribution, with respect to this age categories indicate a shift in the peak of RDT positivity in North Cameroon (uppermost curve) and Kedougou (lowest curve). The middle curve depicts the situation in Adamaoua (Cameroon) which has no history of SMC implementation. This data appears to support SMC expansion to 10 years currently being practiced in Kedougou, Senegal and lends support expanding age of eligibility to 10 years in Cameroon might be beneficial in reducing morbidity. NB. Value labels have not been shown for the Kedougou curve

The overall prevalence of asymptomatic *P. falciparum* infections (API), as defined by RDT + positivity among participants in the study sites is shown Table 3. In Cameroon, the API prevalence in children under five years was highest among study sites in the Nord region of Cameroon, with the lowest prevalence being that in south of Senegal. Table 3 presents API prevalence data stratified by age categories of 5 years in all study sites. There was a general trend of higher carriage in older age groups (5–10 years) in North Cameroon compared to younger age group (0–< 5 years) in all sites. The age distribution of API across the population in each site show similar trend until the age of 40, after which age the trend did not follow a particular pattern and was left out of this age analysis (Fig. 2).

**Table 3:** Prevalence of Plasmodium spp carriage in study populations in Cameroon and Senegal

Site	N	n	%	95%CI
Kedougou (south Senegal)	276	44	15.9	[12.1; 20.7]
Adamaoua	573	218	38.0	[34.2; 42.1]
North	694	231	33.3	[29.9; 36.9]
Adamaoua + North	*1267*	*449*	*35.4*	*[32.8; 38.1]*
All sites	1543	493	31.9	[29.7; 34.3]

N: Number of children aged less than 5 years; n: Number of children aged less than 5 years with a positive malaria test; %: Prevalence of asymptomatic *P. falciparum* infection; 95%CI: 95% confidence interval

When age was analysed as a continuous variable in the general population, the distribution, as shown in Table 4 indicates marked variations between the different sites. In each of the sites, there was a significant difference between the median age of participants who tested positive and who negative for the RDT test. In the study regions in Cameroon, the median age of RDT positive participants was similar—Adamaoua: 8 years, IQR: (4–17), North: 8 years, IQR: (4–12)—although the median age of RDT-negative was lower in North compared to Adamaoua. On the other hand, in Kedougou, Senegal, the median age of RDT positive participants was 11 years [11 years, IQR: (7–16)] and 12 years(IQR: 6–27) among RDT- participants.

**Table 4:** Median age and infection status by RDT test in children and adults in the study sites in Cameroon and Senegal

Site	Country	SMC area	N	Median age RDT_pos Years (IQR)	Median age RDT_neg Years (IQR)	p (Wilcoxon ranksum)
Adamaoua	CAM	No	2240	8 (4–17)	12 (5–25)	p < 0.0001
North	CAM	Yes	2084	8 (4–12)	6 (3–75)	p = 0.0003
Kedougou	SEN	Yes	1773	11 (7–16)	12 (6–27)	p = 0.0009

CAM: Cameroon, SEN: Southern Senegal

### Risk factors for carriage of asymptomatic *Plasmodium* infections in children under five years

Two logistic regression analysis models were developed. The first explored the association between age and asymptomatic carriage of *Plasmodium* spp. as diagnosed by RDT in each of the three study regions and identified confounders. This model considered age as a continuous variable and treated each of the three studies regions independently. The second model explored other factors which might be independently associated with asymptomatic malaria infections in Cameroon, and treated study region as the main exposure variable.

In the first model looking at the association between age and asynptomatic *P. falciparum* carriage (Table 5), it was noted that in Adamaoua age was found to be significantly associated with lower carriage of asymptomatic infections (OR: 0.980, 95%CI: 0.973–0.987, p = 0.000). Similar observations were made when considering gender (marginal association) and if participant had a previous infection in the same year of sampling. Health area, though, was associated with a 11% risk of asymptomatic infections. By contrast, in the North of Cameroon, no significant association between age and carriage of asymptomatic infections was found (OR: 0.998, 95%CI: 0.987–1.008, p = 0.664). A similar finding was made for health area (OR: 1.006, 95%CI: 0.960–1.053, p = 0.808). Having a previous infection in the same year was marginally associated with higher probability of parasite carriage at the time of sampling (OR: 0.887, 95% CI: 0.794–0.992, p = 0.035). In Kedougou, there was a strong association between age and carriage of asymptomatic *Plasmodium* infections (OR: 0.967, 95%CI: 0.967–0.985, p = 0.000) and a similar inference was made for both health area (OR: 0.825, 95%CI: 0.728–0.933, p = 0.002) and whether a participant had a previous infection within the same year of study sampling (OR: 0.563, 95%CI: 0.503–0.631, p = 0.000). These associations were all suggestive of reduced chances of asymptomatic *Plasmodium* infections. No significant influence of gender was noted in the analysis (Table 6).

**Table 5:** Logistic regression table of potential confounding parameters for association of age with asymptomatic *P. falciparum* carriage

Parameter	Adamaoua N = 2197			North N = 2037			Kedougou N = 1676		
	OR	95% CI	p	OR	95% CI	p	OR	95% CI	p
Health Area	1.109	1.059–1.161	0.000	*1.006*	*0.960–1.053*	*0.808*	0.825	0.729–0.933	0.002
Age	0.980	0.973–0.987	0.000	*0.998*	*0.987–1.008*	*0.664*	0.976	0.967–0.985	0.000
Sex	0.834	0.696–0.999	0.049	*0.923*	*0.774–1.102*	*0.376*	0.826	0.647–1.054	0.124
Last infection	0.777	0.713–0.846	0.000	*0.887*	*0.794–0.992*	0.035	0.563	0.503–0.631	0.000

OR = odds ratio. Adults up to 40 years of age in Cameroon and Senegal

**Table 6:** Other determinants associated with *P. falciparum* carriage in Northern Cameroon

Modalities	Univariate analysis			Multivariate analysis		
	cOR	[95%CI]	*p*	aOR (1)	[95%CI]	p
Site						
Adamaoua	1.2	[0.97–1.5]	0.08	1.04	[0.8–1.3]	0.72
North	1			1		
Gender						
Male	1.1	[0.9–1.4]	0.43	1.1	[0.8–1.3]	0.5
Female	1			1		
Presence of bednet						
Present	1			1		
Absent	1.8	[1.2–2.8]	0.004	1.85	[1.2–2.85]	0.005
Duration with bednet						
≥ 2 years	1.4	[1.01–2.1]	0.04	–	–	–
< 2 years	1			–	–	–
Type of bednet						
LLIN	1			–	–	–
Others	4.2	[1.76–11.1]	0.002	–	–	–
Slept under bednet						
No	1.1	[0.7–1.6]	0.7	–	–	–
Yes	1			–	–	–
Bednet bound mat						
Yes	1.1	[0.7–1.6]	0.7	–	–	–
No/other	1			–	–	–
State of the bednet						
< 2 holes	1.1	[0.8–1.5]	0.55	–	–	–
≥ 2 holes	1			–	–	–
Time of last infection						
Last week	1.6	[1.1–2.4]	0.02	1.58	[1.04–2.38]	0.03
Last month	1.7	[1.26–2.3]	< 0.001	1.68	[1.2–2.3]	0.001
This year	1.4	[1.05–1.9]	0.02	1.4	[1.04–1.96]	0.026
Not this year	1			1		
Spent night out of home						
No info	1.01	[0.46–2.1]	0.97	0.6	[0.2–1.5]	0.3
Yes	1.2	[0.76–1.9]	0.4	1.1	[0.67–1.7]	0.7
No	1			1		

– indicate variables which could not be input in the multivariable model due to multicollinearity. This was notable bednet parameters

The second logistic regression analysis concerned Cameroon-only data, with “site” as the main exposure variable. The univariate model shows that being in Adamaoua increased the odds of *P. falciparum* carriage by 1.2 compared to being in the North, but this association appeared not to be significant (p = 0.08). Equally, not having a bed net or having a bed net for more than 2 years or having a bed net other than LLIN, were significantly associated with the carriage of *Plasmodium* infection in children less than 5 years in the Northern Cameroon. In the multivariate analysis, only absence of bed net and having a previous infection in the current year were independently associated with the *Plasmodium* carriage. Other bed net parameters were not included in the multivariate model because of multicollinearity.

## Discussion and conclusion

The primary goal of this study was to determine the prevalence of RDT-confirmed asymptomatic *Plasmodium* infections in a population living in northern Cameroon and southern Senegal where seasonal malaria chemoprevention is practiced or not, and to assess risk factors associated with carriage of infections in children under five years of age. The prevalence of asymptomatic *Plasmodium* carriage among children 3–59 months was found to be lower for southern Senegal compared to Adamaoua and North regions in Cameroon. While in southern Senegal, one in every five children in 2018 had asymptomatic malaria parasite infection, this proportion was almost double in the same group of children in Cameroon. The distribution of *Plasmodium* infections in the study population in different regions indicate a shift in the median age of the group most at risk of infection in the study population. Although this was also observed in Adamaoua, with no history of seasonal malaria chemoprevention exposure, the results may indicate shifting burden of malaria infections to older age groups in Cameroon. In the North region where SMC has been practiced historically, the median age of RDT negative participants was lower and close to the age of eligibility for SMC drug administration. In Kedougou, Senegal, SMC was already being implemented from 2009, in a stepwise fashion, and then scaled up by 2015 among children 3–120 months. In Cameroon, SMC was first implemented in 2016 at scale and has been consistently practiced with SPAQ administered to children 3–59 months since then. Together these observations may point to likely effects of SMC in reducing community carriage of malaria parasites in areas where it is practiced. Indeed, the use of SPAQ in SMC regions has been associated with a broad reduction in parasite biomass in the community [6, 9–11], which in turn has been found to be associated with reduced clinical malaria and mortality during the high transmission seasons in these areas [12, 13].

As data from only a single time-point are presented, this analysis cannot provide evidence of any broad reduction in parasite load in the community following SMC implementation in the North of Cameroon or in southern Senegal. The results do lend further support to the growing body of evidence from Guinea, The Gambia, North Nigeria, Burkina Faso, and Mali, that seasonal malaria chemoprevention with SPAQ leads to significant reduction in the prevalence of *P. falciparum* infection, and thus in mortality and morbidity, in children less than 5 years in SMC areas [6, 9, 12–14]. A recent multi-centre study in seven SMC-implementing countries found evidence of markedly reduced PCR-detectable parasite carriage in under 5 s compared to that in older individuals in the first 2 years following SMC implementation [15]. Consequently, a formal investigation of the likely effects of SMC exposure on community carriage of *Plasmodium* infections as suggested by this baseline study is warranted.

This also suggests asymptomatic infections were less frequent among children eligible for chemoprevention underscoring the importance of adequately addressing malaria in adolescent population as well, as this group may serve as a reservoir of transmission even when infection rates decline substantially in children less than 5 years [16]. Several reports have found transmission of malaria infections is largely driven and maintained by asymptomatic infections [18] and much more among populations out of the range of the SMC group (> 5 years) mostly school aged children [17–21]. A study in The Gambia, however, found no difference in gametocyte carriage comparing rates before and after SMC even though it did not address ongoing transmission in children less than 5 years from the untreated adult population [22]. The small sample size involved may not allow generalizability. While SMC is not primarily deployed as a transmission/reservoir reduction intervention, it has the potential to reduce the residual mass of asymptomatic infections which could lead to onward transmission. A study by Yemeogo et al. in 2021, in which they demonstrated a reduction in human to mosquito transmission of gametocytes through membrane feeds, as well as a negative effect on mosquito longevity; provided some evidence to this effect [23]. In addition, Cissé et al. suggested a reduction in transmission when monitoring the effect of SMC using SPAQ in children up to ten years, conducting entomological monitoring in the same trial [38]. Furthermore, Ahmad et al*.* concluded from a study of asymptomatic carriage of parasites that carriage at the end of a transmission season strongly predicted carriage before in the next [22], suggesting that interventions (such as SPAQ) that clear persistent asymptomatic infections when targeted at the subpopulation with high risk of carriage may reduce the infectious reservoir responsible for launching seasonal transmission in the next season. These few studies form the basis of the suggestion that SPAQ in the context of SMC could have indirect effects which include a population transmission effects.

In the Adamaoua region of Cameroon, the prevalence of asymptomatic malaria was found to be higher in the lower age population (< 5 years) compared to children between 5 and < 10 years, and the trend was different in the North Cameroon and in Kedougou, suggesting a shift in the burden of asymptomatic *Plasmodium* spp carriage away from the 0–5 years age category. This may be related to factors such as differences in transmission at different sites during the season of the survey or possibly on history of exposure to seasonal malaria chemoprevention. It is has been shown that cessation of chemoprophylaxis (in children < 5 years) leads to rebound in older ages (5–10 years) [24]. Given that Adamaoua has no history of SMC exposure, the argument of a rebound might be indicative. However, this remains to be researched. Besides the shifting burden noted in younger age categories, the general trend remains the same in the study population and supports the principle that immunity to malaria develops with age [5]. This observation is similar to a recently reported study carried out in 2015–2017 in Tibati [25], a neighbouring community to Adamaoua study sites, reporting decreasing parasite carriage with age group in both symptomatic and asymptomatic participants in a cross-sectional and hospital-based study. Similarly, Topazian et al., analysing data from a nationally representative demographic health survey, found high parasite prevalence among adults than published rates in children [26]. While the various study designs, parasite detection methods and sampled populations might explain certain small differences between the published studies and our findings, malaria transmission heterogeneities, treatment seeking habits and differences in rates of recent use of interventions might contribute to explain the observed differences in asymptomatic parasite carriage in children and adults [5]. Such areas bordering SMC eligible settings, where malaria seasonality is not marked, may benefit from other chemoprevention strategies such as IPTi or perennial malaria chemoprevention (PMC), as it is now called [27].

### Bed net ownership and use provides additional benefits against asymptomatic malaria infections in Cameroon and Senegal

In Cameroon, the results presented indicate that bed net ownership and the state of the bed net were found to have a significant effect on asymptomatic malaria parasite infection prevalence in Ngaoundere region compared to the neighbouring North region where SMC is implemented. In regions of high malaria transmission, bed net coverage has consistently been found to associate with reduced rates of asymptomatic infections with the malaria parasite. In the Democratic Republic of Congo, as well as in some parts of Cameroon and Senegal, Kenya and Nigeria, this pattern is replicated [28–30]. In studies in Dielmo, Senegal [5], and among adults in Malawi [26], and as found in one of the study sites presented here (North region of Cameroon), bed net ownership or use as well as the state of bed net did not correlate with rates of asymptomatic malaria infections. This may be related to the development of insecticide resistance as has been reported around the study sites [30–32], and in other countries such as in Senegal [16]. In addition, reported bed net use it may not be an accurate indicator of actual use of bed net in the study area, due to reporter bias.

In all sites, those who reported having a previous infection within at least the previous one month had a greater probability of being protected against infection. This could be explained in part by the timing of our survey and the fact that risk of *P. falciparum* infection is heterogeneous and relates to factors, such as bed net use, duration, type, condition, housing materials, and nearness to vector sources, as well as the impact of local climate and vegetation patterns on mosquito abundance. The study described here was carried out a few days prior to the start of SMC drug administration in all sites in North Cameroon and Kedougou, Senegal, making the likelihood of infection from malaria parasite within 28 days less likely. This strongly supports the view that micro-heterogeneity of infection patterns within communities is an important factor in determining individual malaria risk [33–37].

### Age expansion of participants eligible for SMC

The age distribution of asymptomatic *P. falciparum* infection in the current study may suggest that age eligibility for SMC should be reviewed to include children up to 10 years of age. The peak RDT positivity rate appears to be around 10 years for Kedougou, Senegal. Similar pattern is seen in North Cameroon. In Senegal, SMC is administered to children up to 10 years old as a routine practice, which appears to be supported by the results of this study and another study conducted in southern Senegal in 2021 [40]. Studies providing evidence in changes in age patter of infection and clinical disease have been summarized in a systematic review by Caneiro et al., [38] showing that the incidence of severe disease still occurs among the youngest children (under 5 years) although shifts in burden is observed in older children when transmission intensity of malaria falls. Disease severity was not measured in the surveyed populations as this was out of the scope of the study. However, the observation that the burden of asymptomatic infections in children over 5 years is higher in North Cameroun than in Adamaoua with similar malaria transmission intensity may suggest a shift in burden of malaria from younger children (< 5 years) towards older children (> 5 year) in North Cameroon. Further studies are required to clarify if this is due to SMC intervention or other factors affecting transmission in the two settings in Cameroon which might not have been considered in the design of the current study. In Senegal, expansion of age eligibility for SMC was made after analysing data from the first year of implementing the intervention in three health districts in which the authors showed a substantial burden of malaria in children 5–10 years [39]. The decision to expand the age group for SMC implementation will have to take into consideration several other factors. Firstly, it will be important to be sure that covering age groups over 5 years will result in substantial relative decrease in severe disease and deaths. Secondly, it must be shown to be cost-effective and sustainable over the long term and represents an overall cost–benefit to the health system in terms of investments in human resources, procurement of additional medication, systems to monitor and report potential adverse events and the tradeoffs between additional investments to expand SMC age eligibility and investments in other malaria control strategies, such as the use of bed nets among this age group.

This study had some limitations. First, a household survey was conducted to estimate asymptomatic *Plasmodium* infections by RDT test. Blood slide to perform microscopy were not collected due to prevalent logistic challenges. Such an information would have provided more insight into how RDT performed compared to microscopy when estimating proportions of asymptomatic *Plasmodium* infections in field settings. Furthermore, since SMC is implemented in Senegal in children 3 months to 10 years, there is a risk that comparing asymptomatic carriage rates in under-fives versus the general population dilutes the general population with a fraction of children treated. This would be problematic if formal evaluation of the impact of SMC delivery in children 3 months to 5 years was attempted only between the two sites. The goal was to describe baseline asymptomatic carriage rates in SMC settings, prior to administration of SMC doses to children and adults. In addition, examination of the distribution of RDT positivity as a continuous variable or in fine scale age categories, including for children aged 5– < 10 years) show similar overall pattern in North and in Kedougou, Senegal, minimizing an effect of dilution in one subpopulation. Further, there is a risk that sampling in the upper age group (> 50 years) was not done in the same way across the three regions, especially in the North, where fewer old participants were sampled; which may have affected the sample distribution parameters among participants in that region. However, the relatively large sample size of this study allowed an analysis of the variation of RDT positivity up to age 40 years and provided an overall sense of how infections are distributed in children and adults in the three study sites.

Further studies, including using cluster-randomized or case–control before and after designs to permit more robust estimation of the protective effect of SMC and drivers of age-specific asymptomatic carriage of malaria parasites in settings of malaria chemoprevention are recommended. Assessing the levels of molecular markers of SPAQ resistance between sites of differing SMC implementation history is another avenue to explore in future studies to understand the dynamics of parasite persistence or not in the context of malaria chemoprevention.

The prevalence of asymptomatic *Plasmodium* carriage in Cameroon and Kedougou is high (1 in 3 in Cameroon ad 1 in 5 in Senegal) with a shift in infection rates to children 5–10 years which may mirror a potential effect of SMC as seen elsewhere. Bed net ownership was associated with protection and other confounders, such as health area and last infection within the sampling year affected the association between age and carriage in Adamaoua and Senegal, but not in the North Cameroon pointing to heterogeneity in risk of malaria transmission in study sites. Assessing the impact of SMC on malaria incidence and severity using appropriate study designs is needed.

## Competing interests

The authors declare no competing interests.

## Data Availability

Data used to draft the manuscript are contained within. Any other request should be directed to the corresponding author.

## Abbreviations

AQ: Amodiqauine
LLIN: Long lasting insecticide treated bednet
NMCP: National malaria control program
PDA: Portable device assistant
RDT: Rapid diagnostic test
SMC: Seasonal malaria chemoprevention
SP: Sulphadoxine-pyrimethamine
SPAQ: Sulphadoixine-pyrimethamine and amodiaquine
WHO: World health organisation
